# The evolution of the huntingtin-associated protein 40 (HAP40) in conjunction with huntingtin

**DOI:** 10.1186/s12862-020-01705-5

**Published:** 2020-12-09

**Authors:** Manuel Seefelder, Vikram Alva, Bin Huang, Tatjana Engler, Wolfgang Baumeister, Qiang Guo, Rubén Fernández-Busnadiego, Andrei N. Lupas, Stefan Kochanek

**Affiliations:** 1 Department of Gene Therapy, Ulm University, 89081 Ulm, Germany; 2 Department of Protein Evolution, Max Planck Institute for Developmental Biology, Max-Planck-Ring 5, 72076 Tübingen, Germany; 3 Department of Molecular Structural Biology, Max Planck Institute of Biochemistry, 82152 Martinsried, Germany; 4 Institute of Neuropathology, University Medical Center Göttingen, 37099 Göttingen, Germany; 5 Cluster of Excellence “Multiscale Bioimaging: From Molecular Machines To Networks of Excitable Cells” (MBExC), University of Göttingen, Göttingen, Germany; 6 Present Address: Peking-Tsinghua Joint Center for Life Sciences, School of Life Sciences, Peking University, Beijing, 100871 China

**Keywords:** Huntingtin, Huntingtin-associated protein 40, Soluble N-ethylmaleimide-sensitive factor attachment proteins, Retroposition, Single-exon gene, Molecular evolution, Protein coevolution

## Abstract

**Background:**

The huntingtin-associated protein 40 (HAP40) abundantly interacts with huntingtin (HTT), the protein that is altered in Huntington’s disease (HD). Therefore, we analysed the evolution of HAP40 and its interaction with HTT.

**Results:**

We found that in amniotes HAP40 is encoded by a single-exon gene, whereas in all other organisms it is expressed from multi-exon genes. HAP40 co-occurs with HTT in unikonts, including filastereans such as *Capsaspora owczarzaki* and the amoebozoan *Dictyostelium discoideum,* but both proteins are absent from fungi*.* Outside unikonts, a few species, such as the free-living amoeboflagellate *Naegleria gruberi*, contain putative HTT and HAP40 orthologs.

Biochemically we show that the interaction between HTT and HAP40 extends to fish, and bioinformatic analyses provide evidence for evolutionary conservation of this interaction. The closest homologue of HAP40 in current protein databases is the family of soluble N-ethylmaleimide-sensitive factor attachment proteins (SNAPs).

**Conclusion:**

Our results indicate that the transition from a multi-exon to a single-exon gene appears to have taken place by retroposition during the divergence of amphibians and amniotes, followed by the loss of the parental multi-exon gene. Furthermore, it appears that the two proteins probably originated at the root of eukaryotes. Conservation of the interaction between HAP40 and HTT and their likely coevolution strongly indicate functional importance of this interaction.

## Background

Huntingtin (HTT) is a large intracellular protein with a molecular weight (MW) of 348 kDa, which is functionally involved in diverse cellular processes. These include endocytosis, vesicle transport, autophagy, and transcriptional regulation [1]. A mutation in exon 1 of the *HTT* gene, which results in the pathogenic expansion of a polyglutamine tract near the N-terminus of the protein, causes Huntington’s disease (HD), a lethal neurodegenerative disease with autosomal dominant inheritance [2].

HTT orthologs are present throughout protists and animals, but absent in plants and fungi [3, 4]. In mice, HTT is essential for embryonic development and viability, since *HTT* nullizygosity results in early embryonic lethality at about day 8.5 of gestation [5–7]. In zebrafish, *HTT* loss-of-function experiments lead to a variety of phenotypes, including an impact on iron metabolism [8], and different developmental defects, such as impaired neuronal development [9, 10].

Using cryo-electron microscopy (cryo-EM), we recently determined the structure of human HTT [11], which showed a largely alpha-helical protein with three major domains composed of a protein tandem repeat structural motif, the Huntingtin, elongation factor 3 (EF3), protein phosphatase 2A (PP2A), and the yeast kinase TOR1 (HEAT) repeat: a large N-terminal domain with 21 HEAT repeats (N-HEAT), a smaller C-terminal domain with 12 HEAT repeats (C-HEAT), and a connecting bridge domain. Although consisting of homologous repeats, the N-terminal domain forms a one-and-a-half-turn superhelix, whereas the C-terminal domain forms an elliptical ring. Due to the inherent flexibility of HTT [11], structure determination only became possible following the purification of HTT as a tight complex with the cognate huntingtin-associated protein 40 (HAP40). HAP40, formed of 4 canonical and 2 decayed tetratricopeptide repeats (TPR), binds in a cleft between the three domains, forming mainly hydrophobic contacts to N-HEAT and C-HEAT and electrostatic interactions with the bridge domain, thereby stabilizing the conformation of HTT. More specifically, the structure indicated that the C-terminus of HAP40 contains four negatively charged residues (E316, E317, E331, D333), which interact with a positively charged area of the bridge domain. A large number of HTT interactors [1, 12] strongly suggest that HTT serves as a multivalent interaction hub for the coordination of many different functions.

When analysing the interaction of HTT and HAP40 in human 293 cells, HAP40 appeared to be a very abundant interactor of HTT [11]. However, we only obtained the complex between the two proteins upon co-expression, while we could not reconstitute it in vitro from individually purified proteins [11]. The first observation of an abundant, detergent-resistant interaction of HTT with HAP40 in human cell lines was published in 2001 by Peters & Ross [13]. The unusual interaction of HAP40 with HTT, involving coordination of all three HTT domains, explains why in the past HAP40 only rarely surfaced as an interactor of HTT in larger protein-interaction studies unless full-length HTT was used as bait [12, 13]. Interestingly, one of the two studies, which used brain tissue from mice [12], detected HAP40 as the most abundant interactor of HTT, among several hundred less abundant HTT-interacting proteins. These data indicated that the interaction of HTT with HAP40 is not confined to humans.

Very little information on the biological function of HAP40 is available. One research group identified it as an effector of Ras-related protein 5 (Rab5) in endocytosis, mediating the Rab5-dependent recruitment of HTT to early endosomes [14, 15].

In humans, HAP40 is encoded by three sequence-identical paralogs of the factor VIII intronic transcript A (*F8A*) gene (*F8A1*, *F8A2*, *F8A3*) [16, 17], which all are located on the X chromosome at Xq28. While the *F8A1* paralog is contained in intron 22 of the coagulation factor VIII (*F8*) gene, the other two copies are located outside the *F8* gene, closer to the Xq telomere and separated by about 495 kb and 571 kb, respectively, from *F8A1*. The *F8A* genes are single exon genes (SEG), i.e. not containing an intron, and are part of a larger, nearly identical repeat sequence of about 10 kb, named int22h-1, int22h-2, and int22h-3.

The *F8A* genes were first described in the early 1990s [16] when it was noted that recombination between the intra- and extragenic copies of *F8A* results in haemophilia A due to *F8* gene inactivation. This inversion accounts for approximately 50% of all haemophilia A cases [18–20].

The extended interaction of HAP40 with HTT, the stability of the complex during purification, and the abundance of this complex both in human cell culture and in mouse brain [12] indicate that HAP40 is an important HTT cofactor. Since no information has been available about the presence of HAP40 in different species, we analysed the evolution of *F8A* and its potential coevolution with *HTT*. Substantiating functional importance of the HAP40-HTT interaction, our results strongly suggest the involvement of retroposition, i.e. chromosomal integration of reverse-transcribed mRNA, in the generation of the *F8A* SEG in amniotes, the coevolution of *F8A* and *HTT*, and the conservation of the HTT-HAP40 interaction. Further, our results provide evidence for a homologous origin of HAP40 and N-ethylmaleimide-sensitive factor (NSF) attachment proteins.

## Results

### Conversion of F8A from a multi-exon to a single-exon gene during the divergence of amphibians and amniotes

While in humans and mice HAP40 has previously been shown to be encoded by single exon genes (SEGs), with three copies present in humans and only one in mice, we noted early on in our study that in zebrafish (*Danio rerio)* the *F8A* ortholog (zgc:101679) comprises 11 exons and spans about 9200 nucleotides, versus only about 1700 nucleotides in humans and mice. To analyse the emergence of *F8A* SEG from an intron-containing ancestor and its possible co-existence with the parental gene in some species, we analysed the genomic organization and chromosomal localization of the *F8A* locus in 29 representative unikonts (see Additional file 1). Our analyses indicated that *F8A* is a SEG only in amniotes, whereas it comprises more than one exon in all other analysed species. For instance, a multiple-exon organization of *F8A* is present in *Danio rerio* (11 exons), *Xenopus laevis* (12 exons), and *Ciona intestinalis* (18 exons). Moreover, a multiple-exon organization of *F8A* is found in the non-chordates *Amphimedon queenslandica* (8 exons) and *Trichoplax adhaerens* (15 exons). In insects, such as *Drosophila melanogaster* (2 exons) and *Bactrocera latifrons* (2 exons), a smaller number of exons is annotated.

### Copy-number variation and chromosomal location of F8A orthologs

Analysing completely sequenced genomes revealed copy number variation of *F8A* in different species (see Additional file 1). For example, in the order Primates, humans and orangutans (*Pongo abelii*) contain three, chimpanzees (*Pan troglodytes*) and gorillas (*Gorilla gorilla*) contain two, and gibbons (*Nomascus leucogenys*) and white-faced capuchins (*Cebus capucinus imitator*) contain one *F8A* paralog.

Like in the house mouse (*Mus musculus*), only one *F8A* ortholog was detectable in the Norway rat (*Rattus norvegicus*). In contrast, one or two *F8A* orthologs are present in laurasiatherians, such as one copy in cats (*Felis catus silvestris*) and panthers (*Panthera pardus*), and two copies in horses (*Equus caballus*), pigs (*Sus scrofa*), and cattle (*Bos taurus*). In more deeply-branching species, for example in chicken (*Gallus gallus*), zebra finch (*Taeniopygia guttata*), western painted turtle (*Chrysemys picta bellii*), zebrafish (*Danio rerio*), pufferfish (*Takifugu rubripes*), African clawed frog (*Xenopus laevis*), and tropical clawed frog (*Xenopus tropicalis*), we identified only one ortholog.

As noted in the introduction, the three *F8A* paralogs in humans are part of larger repeats (int22h-1, int22h-2, and int22h-3) with a nearly identical sequence. Another SEG, the H2A histone family member B1 gene (*H2AFB1*), is located in the immediate vicinity of *F8A*. *H2AFB1* codes for an atypical and mammalian-specific histone that is associated with the regulation of apoptosis in spermatogenic cells [21], mRNA processing, and active transcription [22]. When analysing the genomic loci of the *F8A* orthologs, we observed that the *F8A* and *H2AFB1* genes co-localise in species with more than one *F8A* gene, such as in human, chimpanzee, orangutan, rhesus monkey, pig, and cattle. In contrast, apart from cats, there is no co-localisation of the *F8A* and *H2AFB1* genes in species with one *F8A* gene (e.g. in gibbon, mouse, and rat). In cats, the *F8A* ortholog at locus LOC101095239 is surrounded by two *H2AFB1* paralogs (LOC101097798 and LOC101098042), although, according to our analysis, cats possess only one *F8A* gene.

When determining the chromosomal localization of *F8A* in 29 representative species (see Additional file 1), we found single-exon *F8A* orthologs to be almost exclusively located on the X chromosome or in X-chromosome-syntenic regions on other chromosomes. On the X chromosome, single-exon *F8A* orthologs were always located close to or inside an intron of the coagulation factor VIII (F8) gene. In chicken, *F8A* is located on chromosome 4 at nucleotides 2,115,165—2,116,487 (GRCg6a), a locus known to be syntenic to the human *F8A* gene and *F8* locus. As the sole exception, in the Norway rat (*Rattus norvegicus*) the *F8A* and *F8* genes are both located on different autosomal chromosomes, namely chromosome 1 and 4, respectively. Our analysis indicates that the genomic localisation of the multi-exon *F8A* is not syntenic to the genomic loci of the single-exon *F8A* orthologs. For instance, the genomic locus of the multi-exon *F8A* ortholog in zebrafish is on chromosome 1 and not syntenic to the human or chicken *F8A* loci. Moreover, in some species such as *Danio rerio*, *Ciona intestinalis, Ciona savigny*, and *Xenopus laevis*, the genomic loci of multi-exon *F8A* orthologs appear to be non-syntenic to multi-exon *F8A* loci in other species. This diversity in gene structure, copy number, and genomic location suggest a complex history for this family.

### HAP40 and HTT are present in all unikonts except fungi

**Fig. 1:**
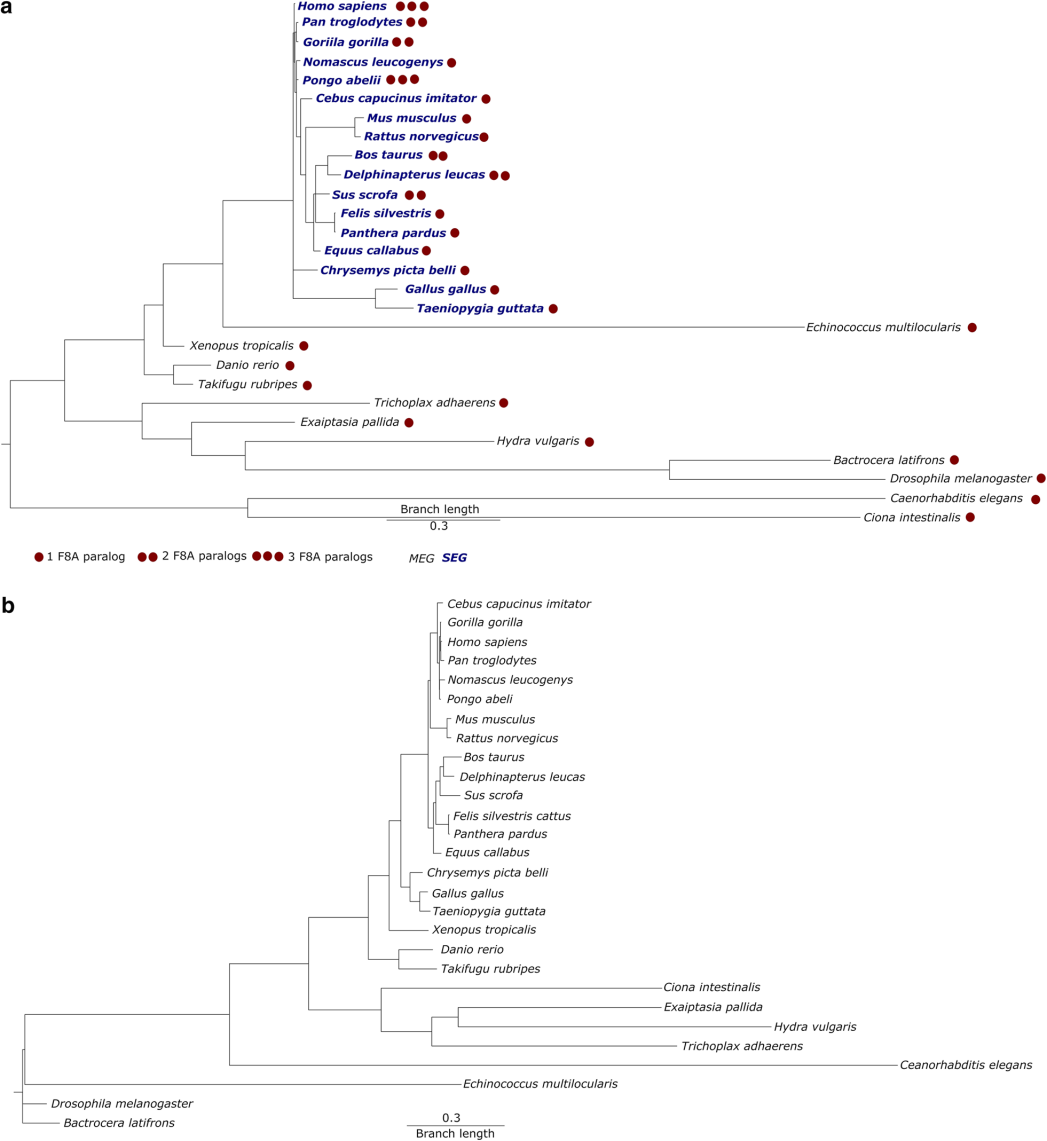
Phylogenetic tree of HAP40 (**a**) and HTT (**b**). The multiple sequence alignments of HAP40 or HTT orthologs were computed using the MUSCLE algorithm [51, 52] implemented in the MEGA X software [53] as described in the methods section. Phylogenetic trees were calculated by Bayesian inference in MrBayes [55] using Yang’s autocorrelated gamma model [56] and a mixed evolutionary model

**Fig. 2:**
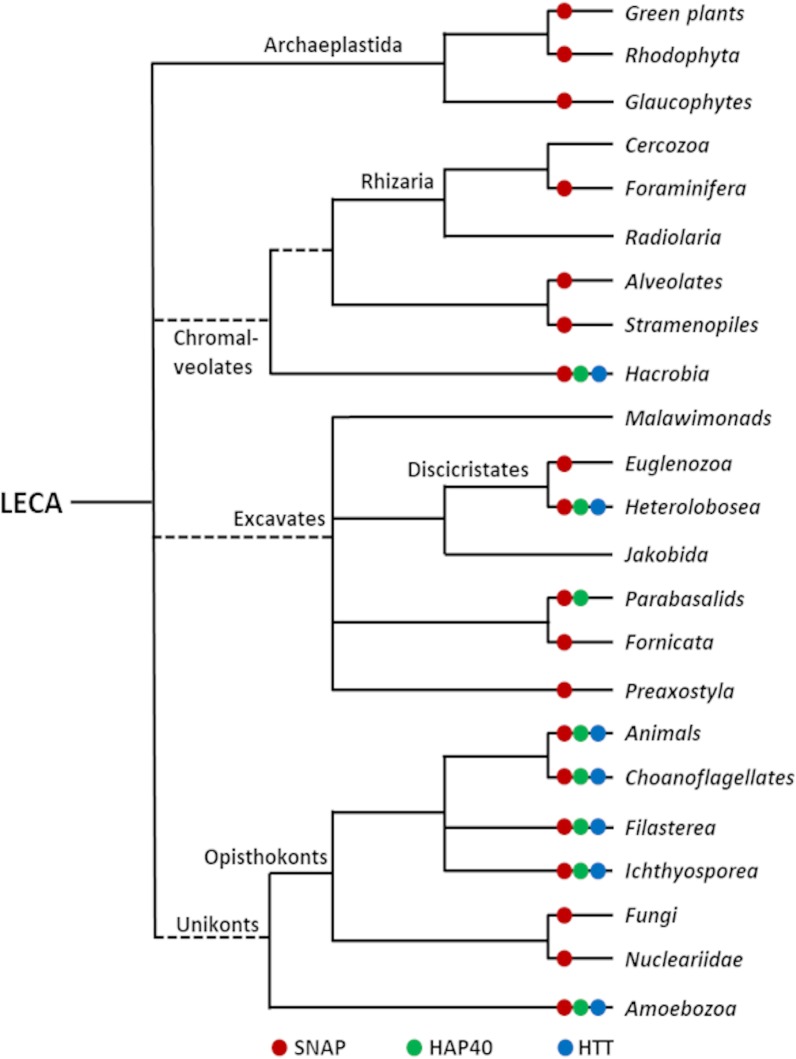
Phylogenetic distribution of HAP40, HTT, and SNAP. HAP40 and HTT are primarily found in unikonts, whereas SNAP is widespread in all eukaryotic lineages. The tree shown here was adapted from the ‘Tree of Life Web Project’ [69]. Branches with uncertain monophyly are indicated by a dotted line

To follow the evolution of HAP40 and HTT, we analysed the non-redundant protein sequence database for their presence either using PSI-BLAST or HHpred. In agreement with published data [23–25]*,* HTT orthologs were found in animals *(e.g. Amphimedon queenslandica* and *Trichoplax adhaerens*)*,* choanoflagellates (e.g. *Salpingoeca rosetta* and *Monosiga brevicollis*), filastereans (e.g. *Capsaspora owczarzaki*), ichthyosporeans (e.g. *Sphaeroforma arctica*), and amoebozoans (e.g. *Dictyostelium discoideum* and *Planoprotostelium fungivorum*), but not in fungi and nucleariids (Figs. 1 and 2, Additional files 2, 3, 4, 5, 6). We conclude that the common ancestor of unikonts contained HTT and that the protein was lost in the lineage leading to fungi. Outside unikonts, we detected potential HTT homologs in one species each of chromalveolates (the cryptophyte *Guillardia theta*) and excavates (the free-living amoeboflagellate *Naegleria gruberi*), but not in archaeplastidans (which include green plants and red algae). Given the very patchy distribution of potential HTT and HAP40 homologs, we cannot judge at present whether HTT and HAP40 originated at the root of eukaryotes, in the Last Eukaryotic Common Ancestor (LECA), and was lost in the plant lineage, or originated in unikonts and was acquired laterally by a small number of other lineages. Strikingly, apart from the parabasalids *Trichomonas vaginalis* and *Tritrichomonas foetus*, in which we only detected HAP40, the presence of HTT and HAP40 correlated perfectly across all organisms, supporting the inference that the two proteins evolved together.

### Conservation patterns of HAP40 and HTT, and their interaction

**Fig. 3:**
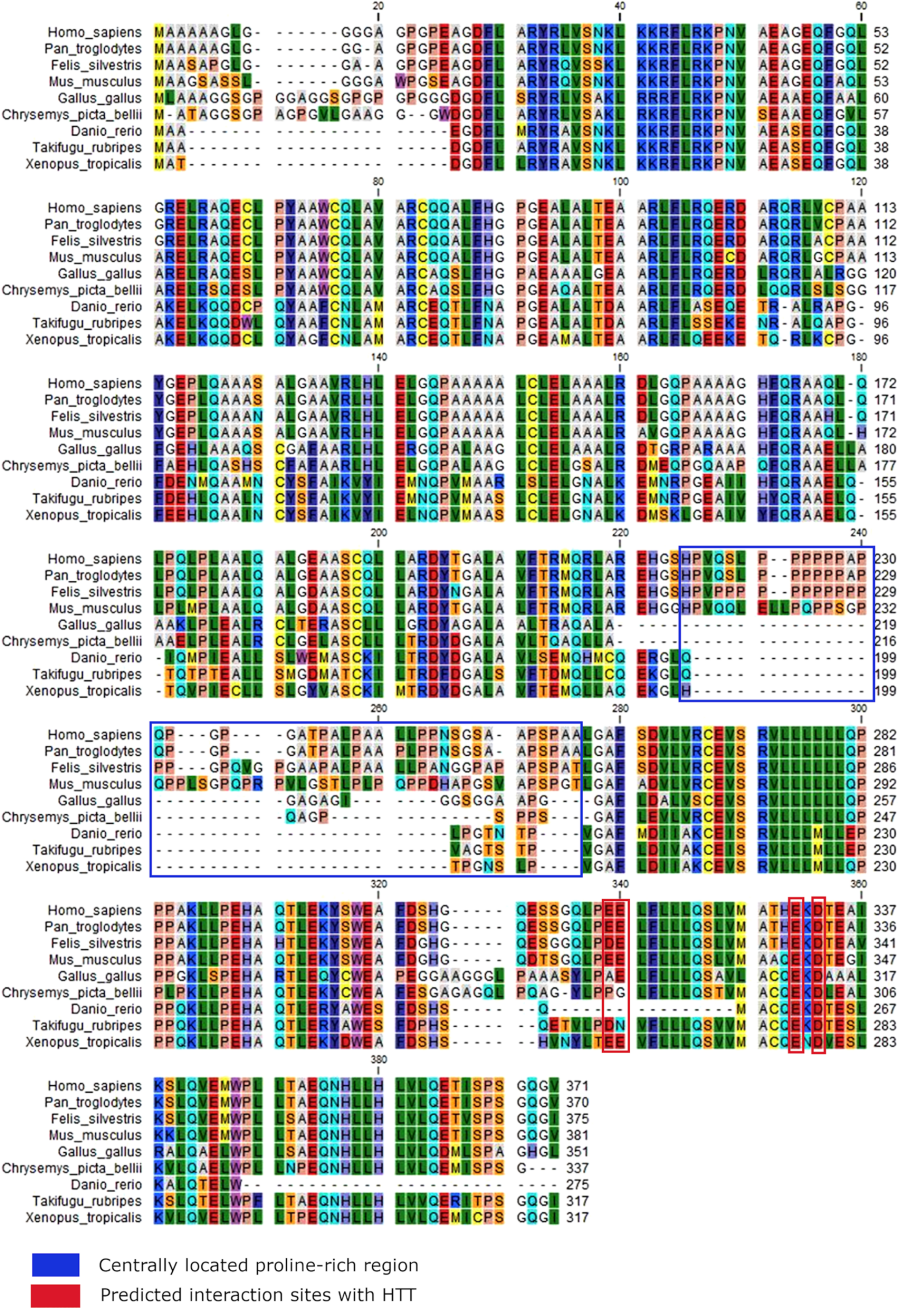
Multiple sequence alignment of HAP40 orthologs from representative vertebrates. The alignment, computed using the CLC Main Workbench 7, illustrates the absence of the centrally located proline rich region in non-mammalian species

**Fig. 4:**
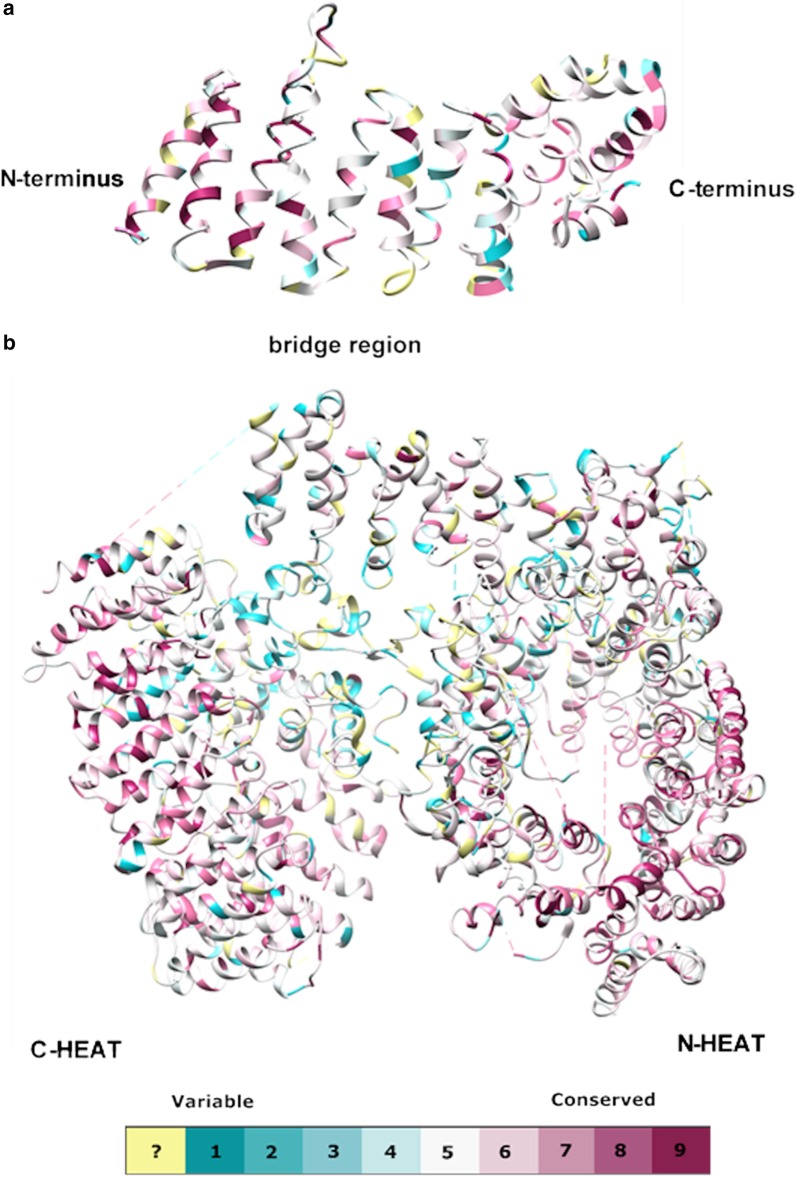
Evolutionary conservation of HAP40 (**a**) and HTT (**b**): Conservation scores were estimated based on a multiple sequence alignment of HAP40 and HTT orthologs from representative species. For the estimation, the ConSurf method [61, 62] with a Bayesian model [4] and the Jones-Taylor-Thornton model [70] was used. Only amino acids that were resolved by Guo et al. [11] (protein database identifier 6EZ8) are shown in the figure

To investigate the conservation patterns of HAP40 and HTT in metazoans, we constructed multiple sequence alignments and mapped their conservation onto the protein structures using the ConSurf server [26]. Alignments of HAP40 from 43 mammals and 73 non-mammals (see Additional file 7) indicated conservation of the N- and C-terminal regions, separated by a variable proline-rich region (41 residues in humans), which is only present in mammals (Fig. 3). Besides, these findings are reflected in the normalized conservation scores computed with the ConSurf method (Fig. 4a, Table 1, Additional files 8, 9, and 12).

**Table 1:** Average conservation score for the different protein domains

Protein	Protein domain	Positions	Conservation score (mean ± SEM)
HAP40	N-terminal domain	1 − 216	− 0.14 ± 0.07
	Central proline-rich region	217 − 258	0.80 ± 0.12
	C-terminal domain	259 − 371	− 0.03 ± 0.09
HTT	N-HEAT	91 − 1684	− 0.09 ± 0.02
	Insertion	400 − 674	0.48 ± 0.06
	N-HEAT without insertion	91 − 399 + 675 − 1684	− 0.20 ± 0.03
	C-HEAT	2092 − 3098	0.35 ± 0.05
	C-HEAT without insertions	2092 − 2120 + 2457 − 2509 + 2664 − 3098	− 0.45 ± 0.03
	Insertion 1	2121 − 2456	0.59 ± 0.06
	Insertion 2	2510 − 2663	− 0.07 ± 0.07
	Bridge	1685 − 2091	− 0.05 ± 0.05

Conservation scores calculated with the ConSurf method [26] as outlined in the method section. Information about the location of HTT domains was taken from Guo et al. [11]. Calculations of the mean and standard error of mean (SEM) were performed using R version 3.5.2 [64] and the R-package readr [66]

In HTT, the N-HEAT domain (residues 91–1684) with 21 HEAT repeats contains a large insertion between repeats 6 and 7 (residues 400–674), which was unresolved in the cryo-EM structure; the C-HEAT domain (residues 2,092–3,098) with 12 HEAT repeats contains insertions between repeats 1 and 2, and repeats 2 and 3 [11]. The two domains are separated by the bridge domain. The ConSurf conservation scores (Additional files 10, 11, 12) show that the insertion in N-HEAT and insertion 1 in C-HEAT are poorly conserved, insertion 2 in C-HEAT and the bridge domain show intermediate conservation, and the HEAT repeats of N- and C-HEAT are the most conserved parts of the protein (Fig. 4b and Table 1).

**Fig. 5:**
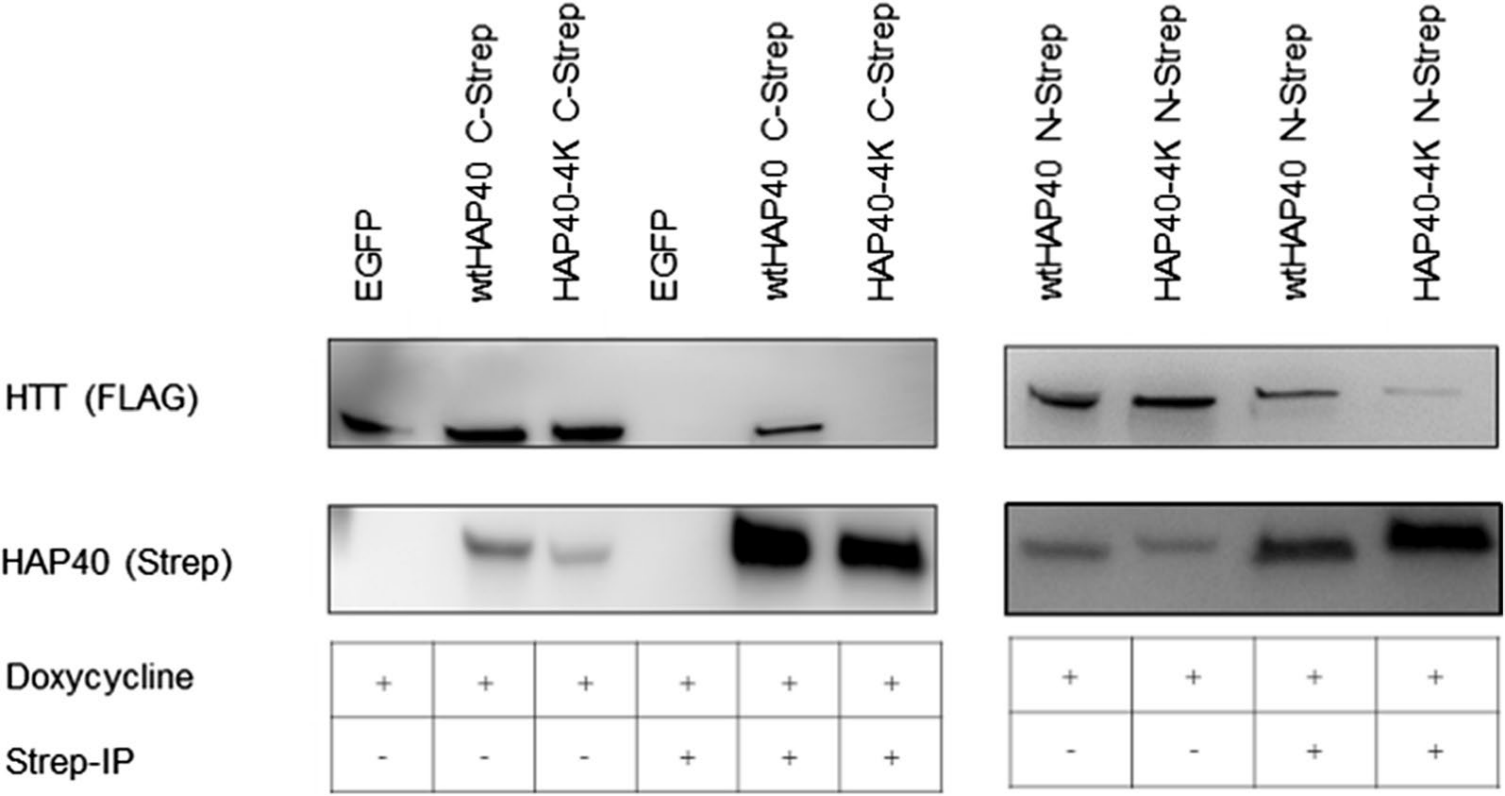
Interaction study of mutated HAP40 and HTT. Human 293-based B1.21 cells, that express 17Q-HTT upon induction, were transfected with plasmids coding for either wtHAP40 or HAP40 mutants in which the E316, E317, E331, and D333 were replaced by lysine (HAP40-4 K). For the interaction study, wtHAP40 and HAP40 fused to a carboxy-terminal and amino-terminal TwinStrep-tag were used. Cell lysates (IP −) or elution (IP +) of the co-immunoprecipitation of wtHAP40 and HAP40-4 K using MagStrep beads (IBA) were analysed by Western-Blot. Western Blots are representative of three independent experiments

In our previous cryo-EM study, we noted that four negatively charged residues in HAP40 (E316, E317, E331, and D333) interact with a positive patch of the HTT bridge domain (K1967, K1968, R1998, R2002, and R2047) [11]. To obtain further information on this interface, we analysed whether mutation of the negatively charged residues of HAP40 inhibits this interaction. We, therefore, performed pull-down assays between wild-type HTT with a poly-glutamine stretch of 17 glutamines (17Q-HTT) and a version of HAP40, in which the four residues were replaced by lysine (HAP40-4 K). Using either HAP40 (Fig. 5) or HTT (data not shown) as bait, our pull-down interaction assays demonstrate an absent or strongly reduced physical interaction between 17Q-HTT and HAP40-4 K fused to a carboxy-terminal or amino-terminal TwinStrep-tag, respectively, in comparison to the wild-type HAP40 (Fig. 5).

Since these data further highlight the importance of the electrostatic interaction between the bridge domain of HTT and the C-terminal part of HAP40, we analysed by bioinformatic methods whether these residues might be evolutionarily conserved. Two interaction patches were suggested by Guo et.al. [11], one formed between the negatively charged E331 and D333 in HAP40 and the positively charged R1998, R2002, and R2047 in HTT, and the second between E316 and E317 in HAP40 and K1967 and K1968 in HTT. In our in-silico analysis, the residues of the first patch were clearly better conserved than average (Table 2) and mostly retained charge complementarity within metazoans, except in insects. In contrast, the residues of the second patch could not be conclusively analysed due to large confidence intervals, but retained charge complementarity in all analysed metazoans, except in *Bactrocera latifrons*, *Amphimedon queenslandica, and Echinococcus multilocularis*. We were unable to explore these potential interactions further based on co-evolution analyses (e.g. with complex [27]) due to insufficient depth of the multiple alignments.

**Table 2:** Conservation of amino acid residues involved in the interaction of HAP40 with HTT

Protein	Amino acid residue	Normalized conservation score	Confidence Interval
HAP40	E316	0.927	− 0.002; 1,599
	E317	0.011	− 0.701; 0.570
	E331	− 1.104	− 1.658; − 0.786
	D333	− 1.219	− 1.658; − 0.955
HTT	K1967	0.169	− 0.574; 0.732
	K1968	0.442	− 0.307,0.946
	R1998	− 0.467	− 1.047; − 0.106
	R2002	1.007	0.122; 1.549
	R2047	− 1.148	− 1.533; − 0.894

Conservation of amino acid residues involved in the interaction of HAP40 with HTT. Results of ConSurf analysis for selected amino acid residues which were postulated by Guo et al. [11] to be involved in the HAP40-HTT interaction

**Fig. 6:**
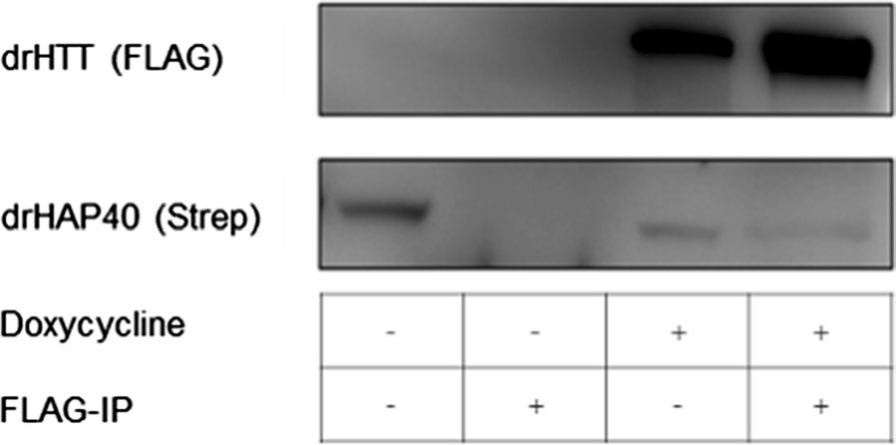
Interaction study of zebrafish HAP40 and HTT. Zebrafish HTT was immunoprecipitated with magnetic anti-FLAG beads from cell lysates of DrHTT-HAP40 cells. The DrHTT-HAP40 cells constitutively express zebrafish HAP40 and the expression of zebrafish HTT can be induced by addition of doxycycline. Cell lysates (IP −) and eluates (IP +) were analysed by Western-Blot analysis using anti-Strep (HAP40) or anti-FLAG (HTT) antibodies. Western Blots are representative of three independent experiments

To determine whether the physical interaction between HAP40 and HTT is also conserved in deep-branching vertebrate species, we performed pull-down assays between HAP40 and HTT from *Danio rerio*. To this end, stable HEK293-based cell lines, co-expressing zebrafish HAP40 and HTT, were generated. Using either zebrafish HTT (Fig. 6) or zebrafish HAP40 (data not shown) as bait, our interaction assays demonstrated a physical interaction between zebrafish HAP40 and HTT. Corroborating the conservation of HAP40-HTT interaction in zebrafish further, we could co-purify zebrafish HTT with zebrafish HAP40 from stably transfected HEK293TetOn cells (data not shown).

Taken together, our bioinformatic analyses and biochemical interaction studies indicate that the physical interaction between HAP40 and HTT is evolutionary conserved at least in vertebrates, but probably throughout animals and, based on the strict co-occurrence of the two proteins, plausibly also throughout unikonts.

### HAP40 and NSF attachment proteins are homologous

Since information on the biological functions of HAP40 is limited, we searched for its homologs among proteins of known structure, using profile hidden Markov models. The search was seeded with HAP40 from human, zebrafish, and fruit fly. The best matches, with probability values > 99%, were the mammalian N-ethylmaleimide-sensitive factor attachment proteins α (SNAPA) and γ (SNAPG), and their yeast ortholog Sec17. SNAP proteins regulate vesicle targeting and fusion by orchestrating the interaction between SNAP receptor proteins (SNAREs) and the cytosolic protein N-ethylmaleimide-sensitive factor (NSF) [28–30]. The SNAP family is widespread in eukaryotes, with many species comprising multiple paralogs; for instance, while yeast contains one homolog (Sec17), humans contain three homologs (SNAPA, SNAPB, and SNAPG) [31].

**Fig. 7:**
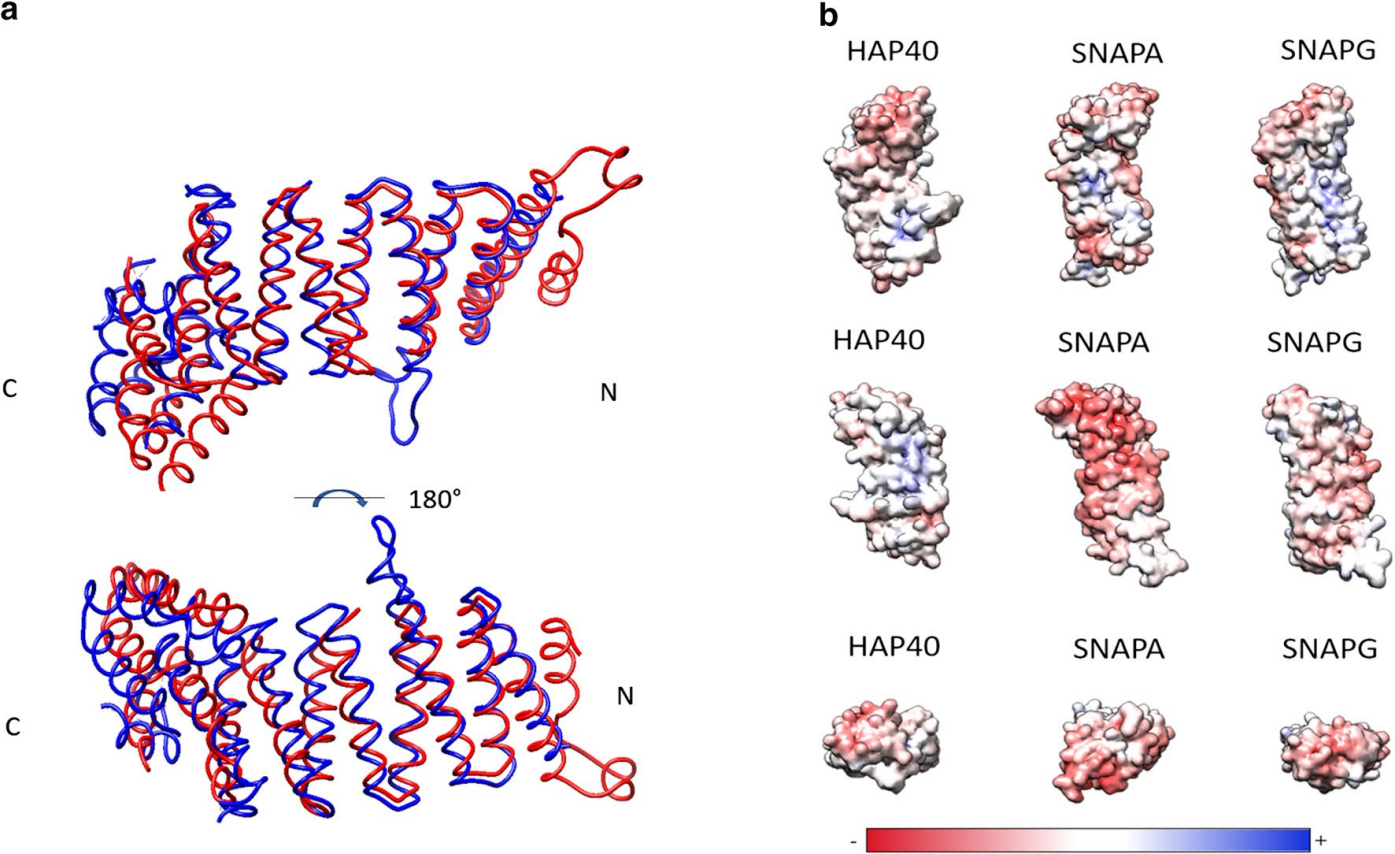
Structure of HAP40 and SNAP proteins. A: Structural alignment of HAP40 (blue, PDB identifier 6EZ8, [11]) with Sec17 (red, PDB identifier 1QQE, [71]), a SNAPA ortholog in *Saccharomyces cerevisiae*, B: Surface charge distribution of HAP40, SNAPA, and SNAPG from different angles in which a red colouring indicates a negative charge and blue indicates a positive surface charge. Illustration of three-dimensional structure was generated with Chimera X in version 1.13.1 [63]

In addition to representing the best hits of HAP40 in sequence space, SNAPs are also its best hits by structure comparison (Fig. 7). The best matches, in a search for HAP40-like structures in the RCSB Protein Data Bank using the DALI webserver [32], were to SNAPA, SNAPG, and Sec17, with Z-scores between 16 and 18, and root-mean-square deviations (RMSDs) between 2.5 Å and 3 Å. For comparison, the next best matches are considerably worse, starting at Z-scores of 13 and RMSDs of 5 Å. Like HAP40, SNAPs are composed of 6 TPR hairpins but lack the insertion in repeat 2 and the proline-rich region found between repeats 4 and 5 in mammalian HAP40.

**Fig. 8:**
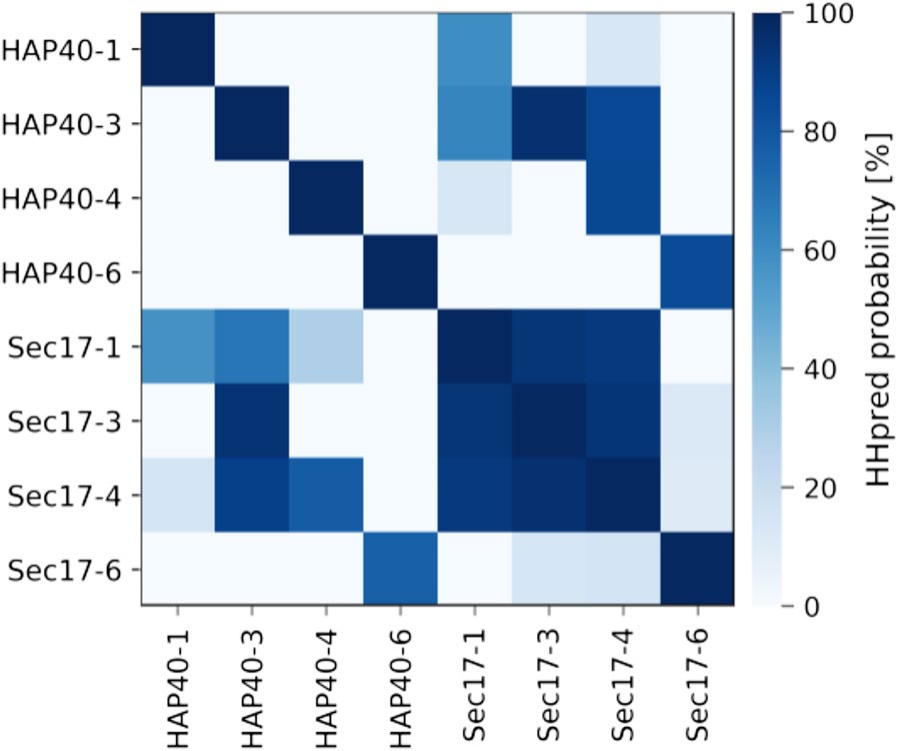
Pairwise HMM comparison of HAP40 and SNAP TPR hairpins. The four canonical TPR hairpins of human HAP40, hairpins 1, 3, 4, and 6, and the corresponding hairpins of the yeast SNAP family protein Sec17 were compared to each other using HHpred

These comparisons show that HAP40 and SNAPs are each other’s closest relatives in protein databases. This homologous relationship could have resulted from the two families having a common ancestor with 6 TPR hairpins or from their independent amplification to similar structures from an ancestral, single TPR hairpin. We have discussed these two scenarios previously [33]. Global sequence similarity in which TPR hairpin *n* of one family matches most closely TPR hairpin *n’* of the other indicates the former scenario, whereas sequence similarity in which all TPR hairpins of one family match each other more closely than any repeat of the other family indicates the latter. Detailed comparisons of the repeats within and between the two families (Fig. 8) show that for the SNAPs there is a clear signal for amplification from a single TPR hairpin since repeat *n* matches the other repeats within the protein with high probabilities. For HAP40, there are no corresponding internal matches, each repeat matching only itself with high probability. Instead, outside the self-match, each HAP40 repeat has its best match to the equivalent repeat of SNAP. This shows that both scenarios mentioned above occurred in the evolution of SNAPs and HAP40: an initial amplification gave rise to the SNAP family, one branch of which differentiated strongly to a new form (HAP40), which therefore has its ancestry in a fully formed SNAP-like TPR protein.

**Fig. 9:**
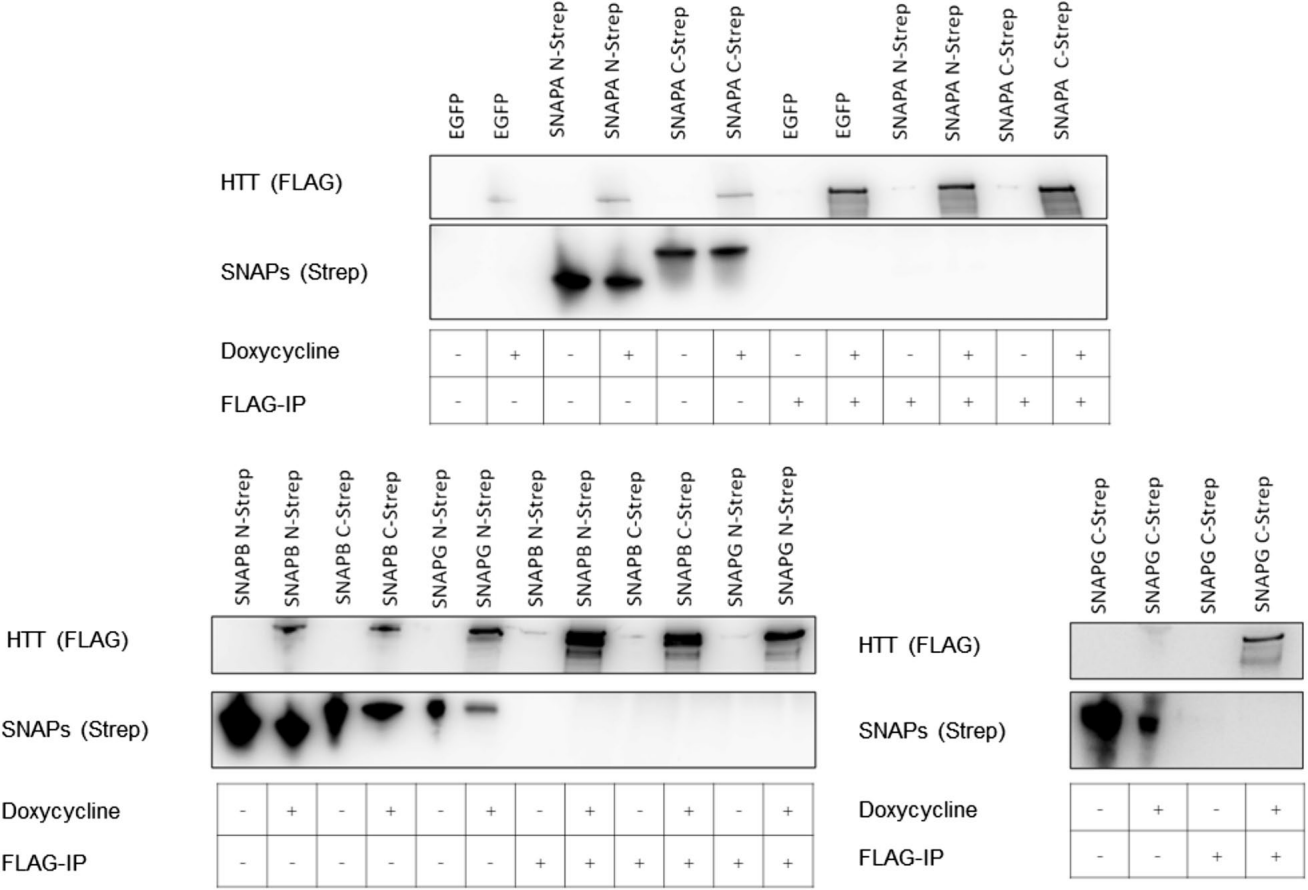
Interaction studies of HTT with SNAPA, SNAPB, or SNAPG using 17Q-HTT as bait. B1.21 cells were induced to express HTT and transfected with pBSK-CMV based plasmids to express SNAPs with carboxy- and amino-terminal TwinStrep-Tags. At 48 h after transfection SNAPs were co-immunoprecipitated using FLAG beads and analysed by western blot. Western Blots are representative of three independent experiments

Based on the observed sequence and structural similarity, we investigated whether the three human SNAP proteins SNAPA, SNAPB, and SNAPG interact with human HTT. In pull-down assays from cell lysates of HEK293TetOn cells, co-expressing 17Q-HTT together with SNAPA, SNAPB, or SNAPG, we could not detect any interaction using either 17Q-HTT (Fig. 9) or the SNAP proteins as bait (data not shown). The absence of a detectable interaction is not surprising, given that SNAPs display a different surface charges distribution than HAP40 (Fig. 7b).

## Discussion

In the human genome, SEGs account for approximately 8.9% of all protein-coding genes [34]. HAP40, a SEG in humans, is expressed from three sequence-identical single-exon paralogs located on the X chromosome, one within and two in the neighbourhood of the *F8* gene. Our analysis of the genomic organization of the *F8A* gene locus across eukaryotes indicated that only amniotes possess the *F8A* gene as a SEG, suggesting that the conversion from a multi-exon gene (MEG) to a single-exon gene (SEG) took place during or early after the divergence of amphibians and amniotes.

Different mechanisms for the emergence of a SEG from a multi-exon precursor have been discussed. According to one, SEGs arise by duplication events in intron-containing genes [35], in which mostly single exons are duplicated generally leading to truncations [36]. This is not the case in *F8A* since the SEG and MEG orthologs code for homologous proteins of nearly identical sizes. Another theory posits that SEGs arise by homologous recombination between a reverse transcript of a mRNA intermediate and the genomic locus of the corresponding gene [37]. Our finding that the *F8A* gene loci in single-exon and multi-exon configurations are not syntenic argues against such a mechanism, although a secondary translocation event cannot be excluded. Retroposition of reverse-transcribed mRNA has been proposed as the predominant mechanism for the generation of functionally active SEGs from parental multi-exon precursors, resulting in retrogenes (also named retrocopies) [34, 38]. Both intron-loss and the observed lack of synteny between the genomic loci of the SEG in amniotes and the MEG *F8A* gene in zebrafish and other non-amniotic species suggest that retroposition of an intron-less gene copy into a new locus occurred, while the original MEG *F8A* locus was lost. The lack of a poly-A sequence does not argue against this mechanism, since retroposition in earlier non-mammalian species, such as chicken [39], seems to be mediated by retroviral mechanisms and not by long interspersed elements which would lead to the integration of a poly-A sequence [38].

Strikingly, primates and some laurasiatherians possess more than one *F8A* paralog, which in humans are contained in a larger int22h repeat. Essentially excluding the possibility that the proteins expressed from the 3 paralogs differ in function, the sequence identity of the three *F8A* paralogs in humans is maintained by gene conversion [19], i.e. a non-reciprocal transfer of genetic material between paralogs due to homology. Most neutral gene duplications have a low probability to become fixed within a population and are frequently lost or evolve to functionally inactive pseudogenes [40]. The duplicated *F8A* genes may have been retained during evolution since the duplication event might have conferred higher evolutionary fitness in human primates and laurasiatherians. However, no data supporting the fixation of the *F8A* duplications have been reported. It is conceivable that for the *F8A* genes either increases in protein expression levels [41] or subfunctionalisation [42, 43] confer evolutionary advantages; the 16S ribosomal RNA [41] and histone genes [40] are examples for a conferred evolutionary advantage by high expression levels due to gene duplications. On the other hand, subfunctionalisation has been hypothesized as the reason for the fixation of highly similar paralogs [40, 43]. For instance, different paralogs might acquire varying tissue- and development-specific expression profiles, as shown for the engrailed-1 and engrailed-1b [40, 42] paralogs. Since the *F8A1* gene is located in intron 22 of the *F8* gene in antisense orientation to F8 transcription, HAP40 expression levels may be influenced by transcriptional activity of the *F8* gene, a gene that is strongly expressed in hepatocytes. However, as an alternative explanation for amplification and fixation of *F8A* paralogs in primates and some laurasiatherians, it is also possible that it is rather the very closely linked *H2AFB1* histone gene that might have conferred an evolutionary advantage, as it has been observed for other histone genes [40].

At the protein level, human HAP40 contains an internal proline-rich region with a length of 41 amino acids that, according to our data, is absent in non-mammals. In mammals, this region is quite variable with respect to length and amino acid composition. This region had remained unresolved by cryo-EM, indicating flexibility, and was dispensable for the interaction of HAP40 with HTT, as shown by co-expression and protein–protein interaction studies in human cells [11]. Frequently proline-rich motifs are directly involved in protein–protein interactions [44] and the identification of interacting proteins binding to this region will be of significant interest to unravel mammalian-specific functions of HAP40.

HAP40 physically interacts with HTT in cells at significant levels, as shown for human cells cultured in vitro [13], and mouse brain in vivo [12]. In this study, we showed a physical interaction between zebrafish HAP40 and HTT, when expressed in human HEK293TetOn cells. We propose that this interaction is evolutionarily conserved, based on an analysis of the interface observed in our cryo-EM structure. Several charged amino acids predicted in that study to mediate the interaction between the C-terminal region of HAP40 (negative charges) and the bridge domain of HTT (positive charges) are conserved in many orthologs, and their mutation in HAP40 decreased the interaction with HTT.

While HAP40 has been suggested to be an effector of RAB5, information on its biological function has largely remained elusive. The recently determined cryo-EM structure of the HTT-HAP40 complex and its thermal unfolding behaviour compared to the individual proteins alone [11] suggest that HAP40 plays a structural role by coordinating the three domains of HTT, shielding the large exposed hydrophobic surface areas that are distributed over a large part of HTT. Since evolutionary relationships can be employed to infer hypotheses about protein functions, we aimed to identify HAP40 homologs with known functions, which might suggest additional functions for HAP40. In our analysis, HAP40 and SNAPs were found to be the TPR-containing proteins with the highest similarity at sequence and structural levels (Fig. 7), raising the possibility that HAP40 could also be involved in vesicular transport. In line with this reasoning, Pal et al. presented data suggesting that HAP40 mediates the recruitment of HTT and the Ras-related protein 5 (RAB5) to early endosomes [14, 15]. Nonetheless, because TPR-containing proteins exhibit highly diverse functions as scaffold proteins [45, 46], it remains unclear to what extent a potential function of HAP40 can be inferred from its homology to SNAPs, all the more since SNAPs cannot replace HAP40 in its interaction with HTT (this study).

## Conclusion

This study is the first analysing the evolution of HAP40, of its encoding gene, the factor VIII intronic transcript A gene (*F8A*) and the potential coevolution of HAP40 with HTT. HAP40 is encoded by a single-exon gene (SEG) in amniotes, whereas it is expressed from multi-exon genes (MEG) in all other organisms. HAP40 co-occurs with HTT in unikonts, including filastereans such as *Capsaspora owczarzaki* and the amoebozoan *Dictyostelium discoideum,* but both proteins are absent from fungi*.* Outside unikonts, a few species, such as the free-living amoeboflagellate *Naegleria gruberi*, contain putative HTT and HAP40 orthologs, raising the possibility that the two proteins evolved at the root of eukaryotes.

The interaction between HTT and HAP40, which was shown in humans and mice, also extends to fish, and bioinformatic analyses provide evidence for the evolutionary conservation of this interaction. The closest homolog of HAP40 in current protein databases is the family of soluble N-ethylmaleimide-sensitive factor attachment proteins (SNAPs). SNAPs, however, are unable to replace HAP40 in the interaction with HTT. Taken together, conservation of the interaction between HAP40 and HTT and their likely coevolution strongly indicate functional importance of this interaction.

## Methods

### Identification of F8A and HTT orthologs in different taxonomic groups

To identify orthologs of *F8A* and *HTT* in different species representing the evolution of eukaryotes, sequence comparisons of the human HAP40 (NP_036283.2) and the HTT (NP_002102) reference sequences were performed by discontiguous megablast, PSI-BLAST [47], or HHpred [48, 49]. For PSI-BLAST, the search was performed against the nr70_euk10Jun (2019) database using human HAP40 (UniProt ID: P23610) as query sequence, the BLOSUM60 matrix, an E-value of 1E−3 and an E-value inclusion threshold of 1E−3. HHpred searches were carried out in the MPI Bioinformatics Toolkit against the PDB_mmCIF70 database using default settings, except for the number of target sequences: 1000. Additionally, we performed searches with HAP40 and HTT orthologs, which were identified by initial searches with the human sequences or by database searches in the protein database of NCBI, from *Drosophila melanogaster, Amphimedon queenslandica*, and *Naegleria gruberi*. We confirmed all identified hits based on their overall sequence similarity, sequence length, and the e-values resulting from the searches with BALST or HHpred, and by visual inspection of global pairwise sequence alignments against the corresponding query sequences using the Needleman-Wunsch algorithm implemented by the European Bioinformatics Institute (EMBL-EBI) [50].

### Calculation of phylogenetic trees

Protein sequences of HAP40 and HTT (Additional file 2) were aligned with the MUSCLE algorithm [51, 52] using the MEGA X software (version 10.0.5, build# 10,180,924-x86_64) [53]. For the MUSCLE algorithm, a gap open penalty of − 2.9 and a gap extend penalty of 0 was used. Moreover, a hydrophobicity multiplier of 1.20 and the unweighted paired-group mean algorithm (UPGMA) with maximal 16 iterations and a minimal diagonal length of 24 was used [51, 52, 54]. Phylogenetic models were calculated by Bayesian inference implemented in MrBayes version 3.2.7.a [55]. For the inference, Yang’s autocorrelated gamma model [56] and a mixed evolutionary model was used. The analysis was conducted for 1,000,000 (HTT) or 500,000 (HAP40) generations on the Baden-Württemberg's high-performance computing cluster (BwHPC). The convergence of phylogenetic trees was determined by the standard deviation of split frequencies which measures the similarity between the tree samples of two independent runs. For HAP40 a standard deviation of split frequencies of 0.011880 and for HTT a standard deviation of split frequencies 0.001884 was reached. Additionally, to test for the robustness of the phylogenetic inference, we computed the phylogenetic trees with the unweighted pair group method with arithmetic mean algorithm (UPGMA) and the maximum parsimony method that are all implemented in the MEGA X software [53] (data not shown).

### Analysis of the F8A gene structure and its genomic locus

The HAP40 gene structure was analysed using the National Center for Biotechnology Information (NCBI) Genome Browser. The chromosomal location and the number of exons were assessed in all selected representative species (Additional file 1), which were chosen to represent the main taxa of unikonts. If the *F8A* gene was not located on the X-chromosome, the *F8A* gene loci were analysed for synteny using the comparative genomics tools provided by Ensembl (release 97) [57]. Since *H2AFTB1* is located in the int22-h repeats in humans and *H2AFTB1* orthologs are only described in mammals, we assessed the localisation of *F8A* in relation to *H2AFTB1* in all selected mammalian species.

In some species (*Amphimedon queenslandica, Bactrocera latrifrons, Caenorhabditis elegans, Ciona intestinalis, Drosophila melanogaster, Echinococcus multilocularis, Exaiptasia pallida, Schistosoma japonicum,* and *Trichoplax adhaerens*), *F8A* orthologues were identified by protein sequence, but not at the nucleotide level by discontiguous megablast using the mRNA sequence from *Homo sapiens* or *Xenopus tropicalis*. Since some *F8A* copies might be not annotated, we excluded these species from the analysis of the number of gene copies to avoid the influence of incomplete gene annotation on our results.

### Presence of the central proline-rich region of HAP40 in different orthologs

To assess the presence of the central proline-rich region of HAP40 orthologs in all representative species, searches in the protein database of the NCBI were performed with protein–protein Basic Local Alignment Search Tool (BLAST) against the metagenomics protein database provided by the NCBI. The amino acid sequence of human HAP40 (accession number: NP_036283.2) was used as the query sequence. To perform an in-depth analysis of the major taxonomic groups of mammals and non-mammals, we analysed HAP40 sequences from species belonging to common orders of mammals and classes of non-mammals. Therefore, sequences of 43 mammals and 73 non-mammals (see Additional file 7) were analysed by pairwise local sequence alignment with the Smith-Waterman algorithm against the human reference sequence [58] using the pairwise sequence alignment tools of the European Bioinformatics Institute [59] and the Blocks Substitution Matrix 50 [60]. The presence of the central-proline rich region was checked by manual inspection of the resulting alignments.

### Estimation of evolutionary conservation with the ConSurf method

Conservation scores were calculated by the ConSurf method [26, 61, 62] based on a multiple sequence alignment of representative HAP40 or HTT orthologs (see Additional files 8, 9, 10 and 11) computed by the MUSCLE algorithm as explained in the section “calculation of phylogenetic trees”. Conservation scores were normalized to a standard deviation of 1 and a mean of 0 [61]. Negative conservation scores indicate higher evolutionary conservation in comparison to other residues of the same protein. The calculated conservation scores were projected onto the HTT-HAP40 protein structure described by Guo et al. [11] (PDB database identifier 6EZ8). Molecular graphics and analyses were performed with UCSF Chimera (version 1.13.1), developed by the Resource for Biocomputing, Visualization, and Informatics at the University of California, San Francisco, with support from NIH P41-GM103311 [63].

Analyses of conservation scores were performed using the R scripting language [64] and the R-packages ggplot2 [65] and readr [66]. The arithmetic means and standard errors of the mean of the conservation score of the amino acid residues in a certain protein domain were calculated (Additional file 12). The positions of the analysed HTT domains were taken from Guo et al. [11].

### HMM-based comparison of HAP40 and SNAP TPR hairpins

To investigate the evolutionary origin of the HAP40 and SNAP families**,** we evaluated the sequence similarity of their TPR hairpins. We chose the four canonical TPR hairpins of human HAP40, hairpins 1, 3, 4, and 6, and their corresponding hairpins in the yeast SNAP family protein Sec17 as representatives. We first searched the nr70 database (NCBI non-redundant protein sequence database clustered at 70% sequence identity) for homologs of human HAP40 and yeast Sec17 using BLAST [47, 67], with E-value threshold (-evalue) set to 0.001 and alignment coverage (-qcov_hsp_perc) to 50%. Separate multiple sequence alignments of HAP40 and Sec17 homologs were parsed out from the obtained hits. These two alignments were subsequently used to extract alignments of the individual TPR hairpins. Profile hidden Markov models (HMMs) were computed from the alignments using hhmake and compared with hhsearch (secondary structure scoring was switched off), both from the HH-suite3 software package for sensitive sequence searching based on HMMs [49].

### Interaction studies with mutated human HAP40 and 17QHTT

The HEK293TetOn-based cell line B1.21 [68] was induced for 72 h with 1 µg/ml doxycycline and transfected, using polyethyleneimine, with pBSK-CMV based plasmids coding for wtHAP40 and HAP40-4 K (p.[Glu316Lys;Glu317Lys;Glu331Lys; Asp333Lys]) with either a carboxy- or amino-terminally fused TwinStrep-tag. As a control, B1.21 cells were transfected with a plasmid coding for EGFP. Cells were collected with DPBS followed by centrifugation for 10 min at 400×*g*. Cell pellets were lysed with 25 mM HEPES, 300 mM NaCl, 0.5% Tween 20, protease inhibitor (Roche), pH 8.0 for 30 min. Lysates were cleared by centrifugation for 10 min at 20,000×*g* and incubated at 4 °C or 3 h with either MagStrep beads (IBA) or magnetic anti-Flag beads (Sigma Aldrich). Beads were washed three times with 25 mM HEPES, 300 mM NaCl, 0.02% Tween 20, pH 8.0. Bound proteins were eluted with pre-heated SDS loading buffer. Samples were analysed with SDS-PAGE and Western blot analysis using anti-Flag (Sigma Aldrich, 1:2500 dilution) and anti-Strep antibodies (IBA, 1:1000 dilution).

### Generation of a stable cell line for co-expression of HTT and HAP40 from zebrafish

A cDNA, human codon-optimized and coding for full-length zebrafish HTT (NCBI NP_571093) and c-terminally fused to a FLAG-His affinity tag, was generated by DNA synthesis (Thermofisher). The cDNA was cloned into plasmid vector pTRE-Tight-BI-AcGFP1 (Clontech) allowing for co-expression of zebrafish-HTT and GFP upon induction with doxycycline (Dox). The resulting plasmid was verified by restriction analysis and transient expression in 293 cells. HEK293 Tet-ON cells (Clontech) were co-transfected with the linearized expression plasmid and a linearized selection plasmid coding for a hygromycin resistance gene. Positive cell clones were isolated by hygromycin selection. A monoclonal cell line expressing zebrafish-HTT (drHTT) was obtained by limited dilution and was validated by Western Blot analysis of cell lysates using a monoclonal anti-FLAG antibody (Sigma Aldrich).

Expression plasmid pBSK/CMV-drHAP40TS was constructed to express, under control of the hCMV promoter, human codon-optimized zebrafish HAP40 (NCBI XP_005160094.1) fused to a c-terminal Twin-Strep-tag. drHTT cells were transfected with pBSK/CMV-drHAP40TS together with a plasmid coding for puromycin resistance. Puromycin-resistant cell clones were isolated and a monoclonal cell line (drHTT-HAP40 3-2-26) was derived by limited dilution. drHTT-HAP40 3-2-26 cells were validated by Western blot analysis of cell lysates with a monoclonal anti-FLAG ab (Sigma Aldrich, 1:2500 dilution) for detection of Dox-inducible drHTT expression, and an anti-Strep antibody (Iba, 1:1000) for detection of constitutive drHAP40 expression.

### Interaction studies with HAP40 and HTT orthologs from Danio rerio

The HEK293-based cell line zHTT-zHAP40 were induced for 72 h with 1 µg / ml doxycycline. Not induced zHTT-HAP40 cells were used as control. Pull-down assays were performed as described before.

### Interaction studies of SNAPA, SNAPB and SNAPG with human wild-type HTT

Previously described HEK293TetOn-based B1.21 cells, expressing 17QHTT upon induction with doxycycline [68] were transfected with pBSK-CMV-based plasmids expressing human SNAPA, SNAPB, or SNAPG with either carboxy- or amino-terminally fused Twin-Strep tag. Pull-down assays were performed as described before using FLAG beads.

## Supplementary information

**Additional file 1.** f8a orthologs in different species. Genes identified by discontiguous megablast using either the f8a from Homo sapiens (NM_012151.3) or Xenopus tropicalis (NM_001078703.1).

**Additional file 2.** Accession numbers of identified HAP40 and HTT orthologs.

**Additional file 3.** Alignment of HAP40 orthologs used for the phylogenetic analysis.

**Additional file 4.** Phylogenetic tree of HAP40 orthologs.

**Additional file 5.** Alignment of HTT orthologs used for the phylogenetic analysis.

**Additional file 6.** Phylogenetic tree of HTT orthologs.

**Additional file 7.** Species used to analyse the presence of the central proline-rich region in HAP40. Protein sequences of HAP40 orthologs from representative mammalian and non-mammalian species were analysed by local pairwise-sequence alignments.

**Additional file 8.** Alignment of HAP40 orthologs used for the ConSurf analysis.

**Additional file 9.** Raw result of ConSurf analysis for HAP40.

**Additional file 10.** Alignment of HTT orthologs used for the ConSurf analysis.

**Additional file 11.** Raw result of ConSurf analysis for HTT.

**Additional file 12.** R script used for the computation of the domain conservation.

## Data Availability

All analysed nucleotide and protein sequences can be retrieved from the nucleotide or protein databases of the National Center for Biotechnology Information (NCBI) with the accession numbers given in the additional files. Moreover, alignments used for the computation of phylogenetic trees and sequence conservation were added to the additional files.

## Abbreviations

F8: Coagulation factor VIII
F8A: Factor VIII intronic transcript A
GFP: Green fluorescent protein
H2AFB1: H2A histone family member B1 gene
HAP40: Huntingtin-associated protein 40
HD: Huntington’s disease
HEAT: Huntingtin, elongation factor 3, protein phosphatase 2A and lipid kinase TOR
HHpred: Homology detection and structure prediction by HMM-HMM comparison
HTT: Huntingtin
LECA: Last Eukaryotic Common Ancestor
MEG: Multi-exon gene
NSF: N-ethylmaleimide-sensitive factor
PSI-BLAST: Position-specific iterative basic local search alignment tool
RAB5: Ras related-protein 5
SEG: Single-exon gene
SNAPA: Soluble N-ethylmaleimide-sensitive factor attachment protein α
SNAPB: Soluble N-ethylmaleimide-sensitive factor attachment protein β
SNAPG: Soluble N-ethylmaleimide-sensitive factor attachment protein γ
SNAPs: Soluble N-ethylmaleimide-sensitive factor attachment proteins
TPR: Tetratricopeptide-like helical domain
